# Supplementary material for: Synchronization between peripheral circadian clock and feeding-fasting cycles in microfluidic device sustains oscillatory pattern of transcriptome
Source: Nat Commun. 2021 Oct 26;12:6185. doi: 10.1038/s41467-021-26294-9 (PMC8548598; doi:10.1038/s41467-021-26294-9)
Supplement: Supplementary file 1 — Supplementary Information [file 41467_2021_26294_MOESM1_ESM.pdf]

Suppl. Fig. 1

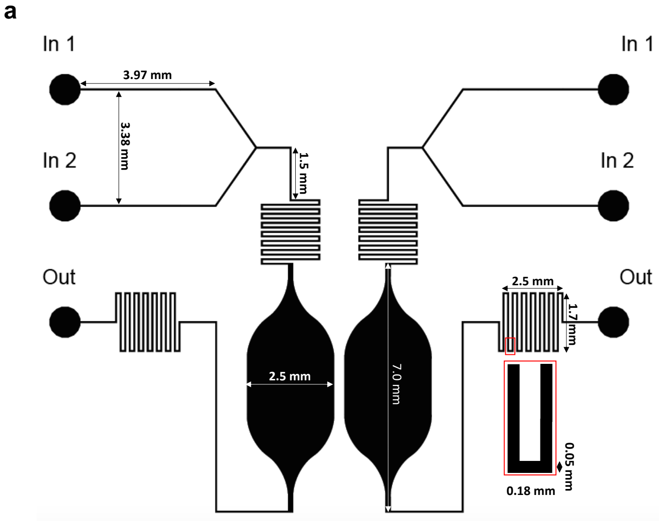

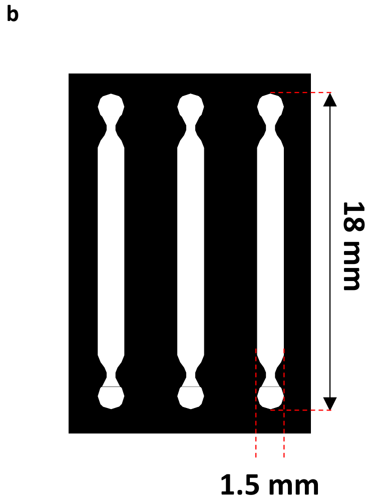

Supplementary Figure 1: Microfluidic platforms. (a) Photomask of the microfluidic device n.1 with the geometric details, designed with CAD; the microfluidic chip is composed of 2 parallel independent chambers with a volume of 1.75 uL each. (b) Photomask of the microfluidic device n.2, with the geometric details, designed with CAD; the microfluidic chip is composed of 3 parallel independent channels with a volume of 2.7 uL each.

Suppl. Fig. 2

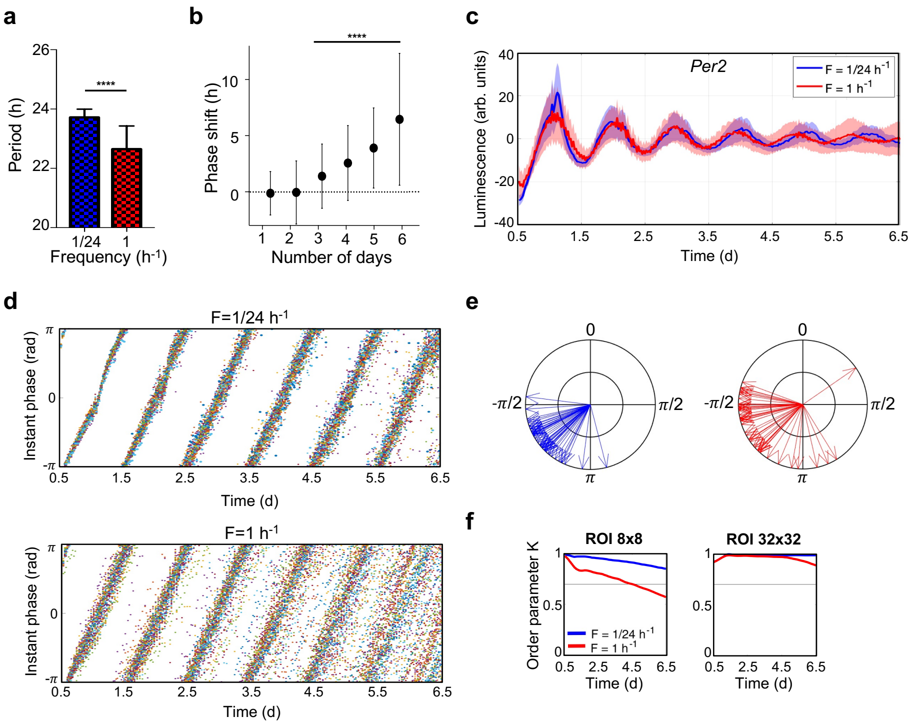

Supplementary Figure 2: Characterization of Per2 oscillations by luminescence in microfluidics. (a) Comparison of the period between the two conditions. Data are represented as mean ± s.d. of the 48 ROIs indicated in Figure 1h. ****P<0.0001 two-sided Student's t-test. (b) Comparison of the phase shift between the two conditions. Data are represented as mean ± s.d., of the 48 ROIs indicated in Figure 1h. ****P<0.0001 one-way ANOVA with Tukey's multiple comparisons test. (c) Per2::Luc 6-day bioluminescence signal intensity in the 16-by-16 pixel ROIs, shown in the scheme in Figure 1h, under the two medium change conditions. The plot is equivalent to the one reported in Fig. 1f (bottom), except for the fact that signal baseline was subtracted based on the average of a centered moving window of 24 hours. Solid line: mean signal intensity, patch: area delimited by mean+/- s.d. of the 48 ROIs indicated in Figure 1h. (d) Instantaneous Hilbert phase of the mean Per2::Luc 6-day baseline-subtracted bioluminescence signal intensity in each of the 16-by-16 pixel ROIs indicated in Figure 1h, under the two medium change conditions indicated in Figure 1f. (e) Day-3 instantaneous Hilbert phase of the mean Per2::Luc bioluminescence signal intensity in each of the 16-by-16 pixel ROIs indicated in Figure 1h, under the two medium change conditions indicated in Figure 1f. (f) Kuramoto order parameter K, calculated for the time intervals between time 0.5 d and the time shown on the x-axis, indicating the level of synchrony between ROIs of different size (8-by-8 and 32-by-32 pixel, as indicated), covering the same area in the chamber schematically shown in Figure 1h. The gray line highlights a value of 0.7.

Suppl. Fig. 3

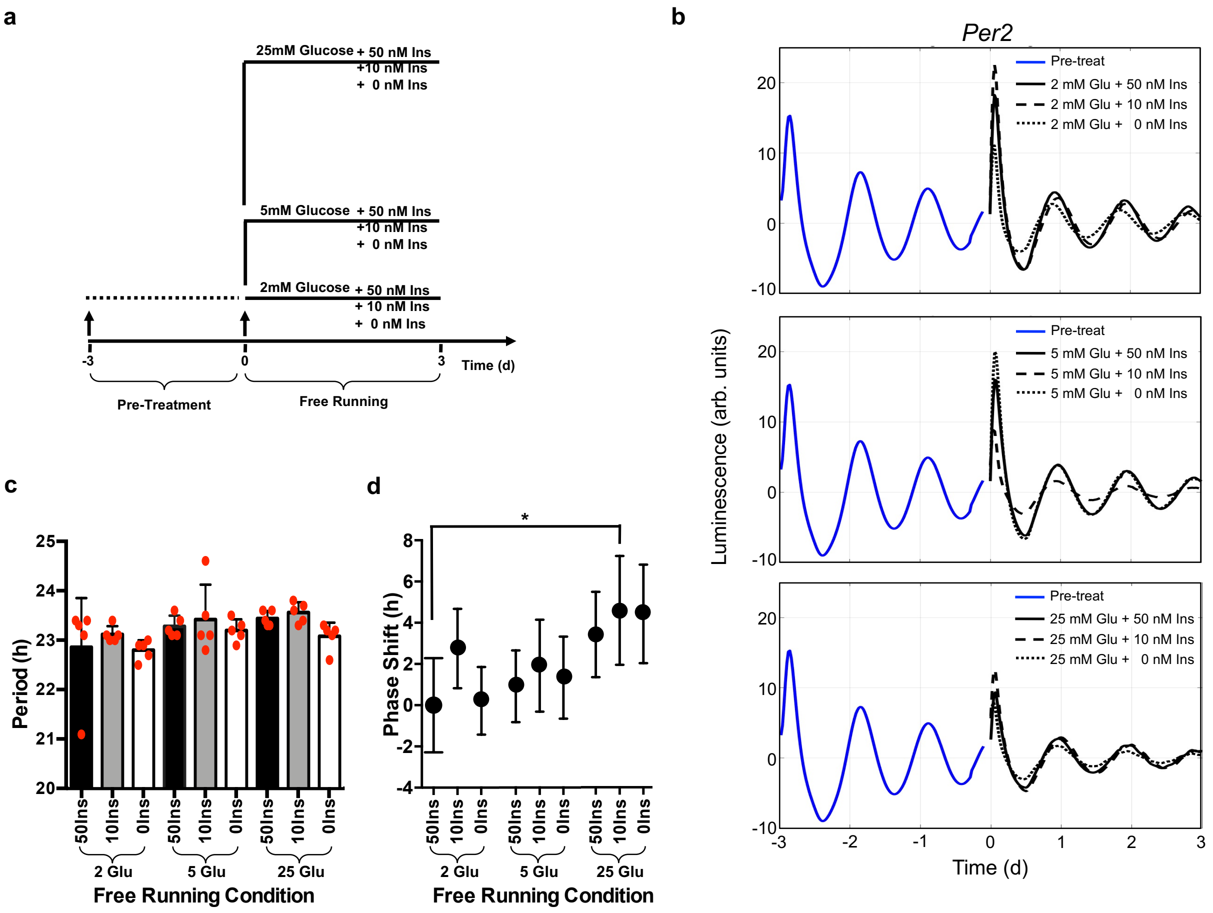

Supplementary Figure 3: The circadian entrainment through glucose-insulin pulse of Per2 fibroblasts. (a) Schematic representation of synchronization protocols imposed at different combinations of glucose and insulin. (b) Baseline-subtracted Per2::Luc bioluminescence rhythms acquired during the pre-treatment in LOW content of glucose and insulin and Free Running (t=0) at intervals of 30 min; data are represented as mean of the bioluminescence signal, N=5 for each group. (c) Comparison of the circadian period for the different metabolic perturbations at FR. Data are represented as mean ± s.d., N=5 for each condition. (d) Phase of all conditions; the phase is calculated at the second peak observed after t=0, using 2 mM Glu+50 nM Ins as control. *P=0.05376, one-way ANOVA with Tukey's multiple comparisons test. Data are represented as mean ± s.d., N=5 for each condition.

Suppl. Fig. 4

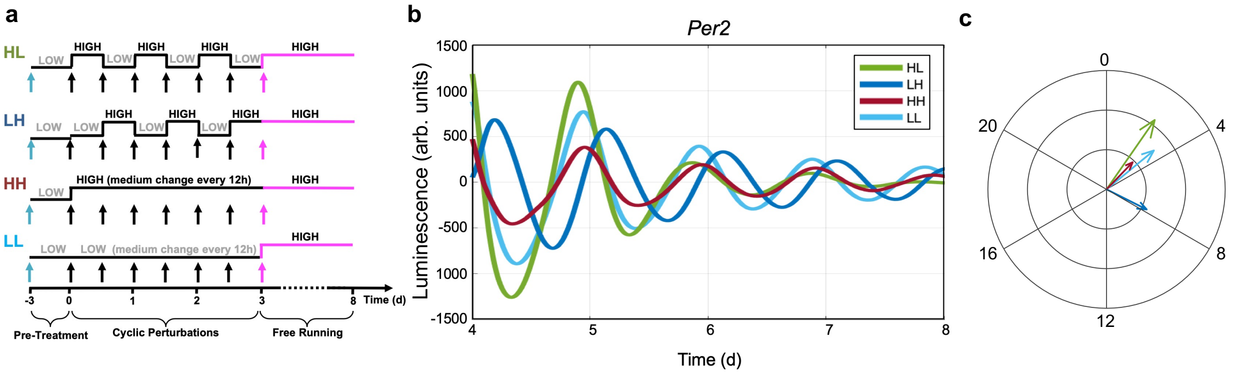

Supplementary Figure 4: Metabolic behavior of cyclic day and night in conventional dish. (a) Schematic representation of the 4 metabolic perturbations, by alternate cycles of high and low glucose and insulin levels (H= 25 mM - 100 nM and L= 2 mM - 0 nM, respectively). The 24h cyclic perturbations implemented before the Free Running (FR), in L condition, are: HL (12 h of H and 12h of L), LH (12 h of L and 12h of H), HH (12 h of H and 12h of H) and LL (12 h of L and 12h of L); all perturbation cycle conditions present a starvation pre-treatment, in L condition, for 3 days; the recording is performed in H condition (FR). (b) Baseline-subtracted Per2::Luc bioluminescence patterns acquired after 3 days of stimulations. Data are represented as mean of bioluminescence signal, N=3 for each condition. (c) Polar representations of the relative temporal position of the acrophases of HL, LH, HH and LL between day 4.5 and 5.5 of FR, N=6 for each group; the arrow length (radial axis) indicates the value of amplitude, the angular position indicates the acrophase. The variations of the acrophase expressed as standard deviation are 0.58 h (HL), 0.51 h (LH), 0.1 h (HH), 0.29 h (LL).

Suppl. Fig. 5

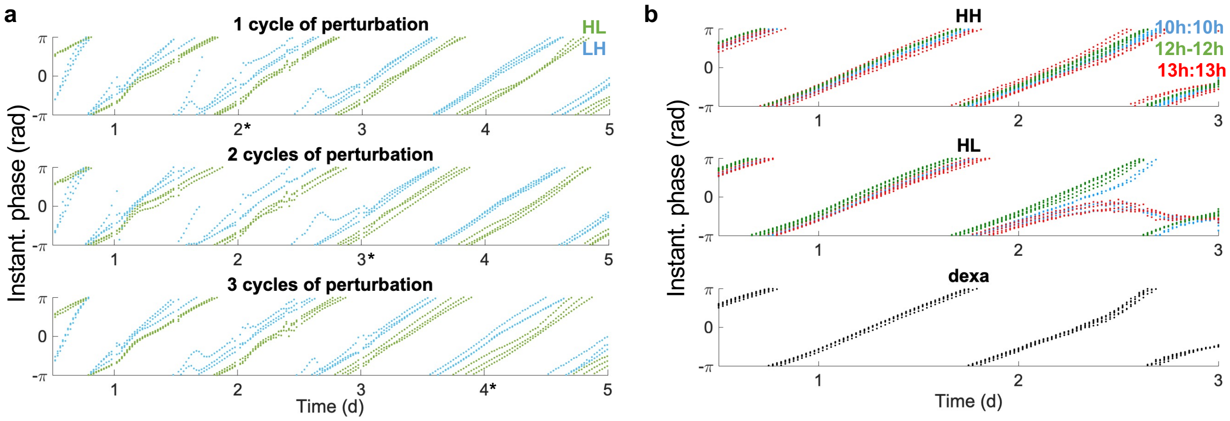

Supplementary Figure 5: Phase analysis of feeding-fasting patterns imposed at different periods. (a) Instantaneous Hilbert phase of the baseline-subtracted mean Per2::Luc bioluminescence signal intensity acquired in the experimental conditions HL and LH reported in Fig. 3a-b; each dot represents the signal from one ROI at each time point that corresponds to the surface of one channel N=3 for each condition. The (*) symbol indicates the second day of FR for each condition, at which the relative temporal position of the acrophases was calculated in Fig. 3c. (b) Instantaneous Hilbert phase of the baseline-subtracted mean Per2::Luc bioluminescence signal intensity acquired in the experimental conditions acquired reported in Fig. 3e; each dot represents the signal from one ROI at each time point that corresponds to the surface of one channel N=4-6 for each condition.

Suppl. Fig. 6

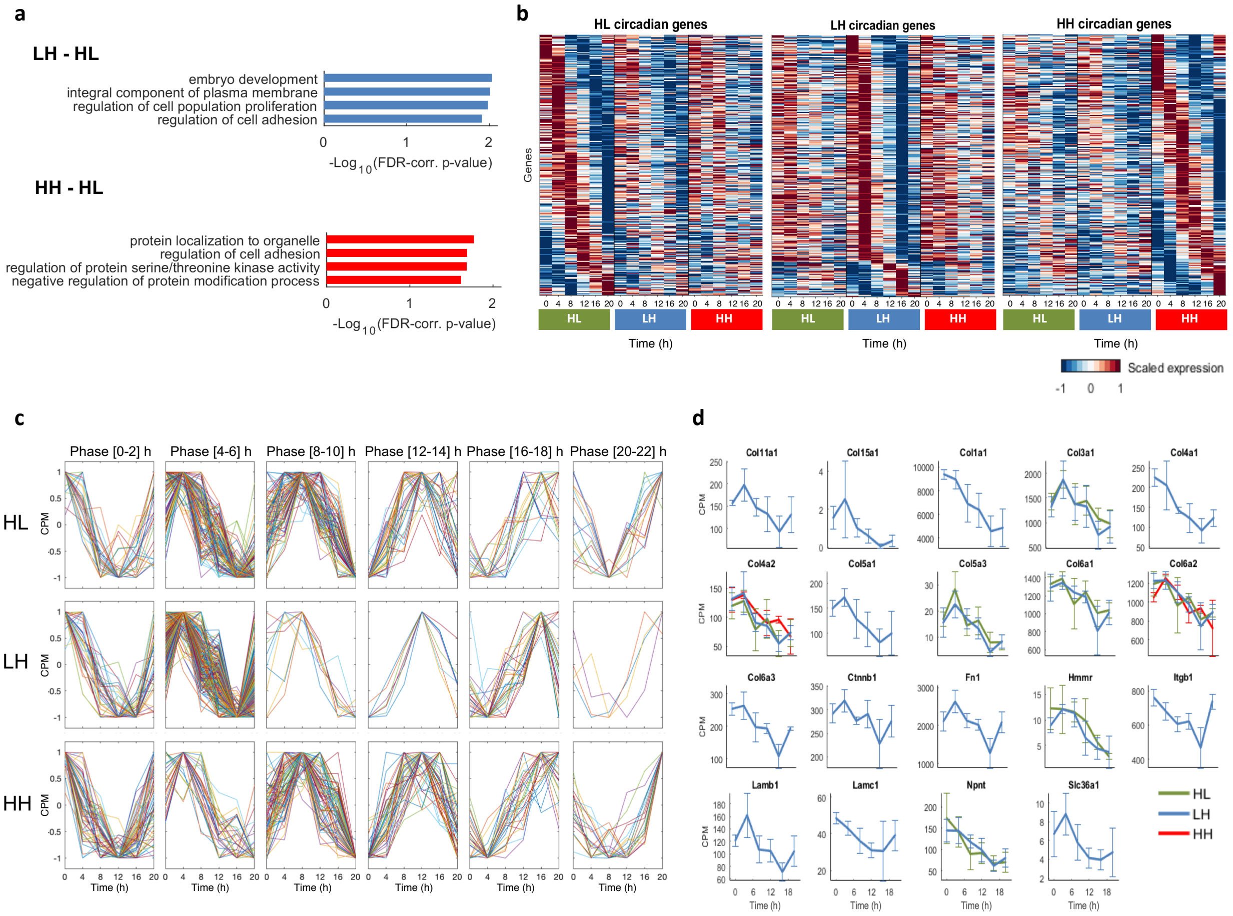

Supplementary Figure 6: Gene ontology enrichment of circadian oscillating transcripts. (a) Enrichment analysis of genes that are circadian in both the indicated conditions but have an absolute oscillation phase difference higher than 2h. All three GOs (GO-BP, CC, and MF) were searched simultaneously. Only the most relevant categories are shown, the complete results are reported in Supplementary Spreadsheet S1. (b) Heat maps of genes circadian under each of the conditions indicated in the respective sub-figure titles. (c) Circadian gene profiles clustered by phase in HL (top), LH (middle) and HH (bottom). (d) Temporal profiles of ECM genes only in the indicated conditions where they are oscillating ($p < 0.01$ of JTK test); data are represented as mean ± s.d.

Suppl. Fig. 7

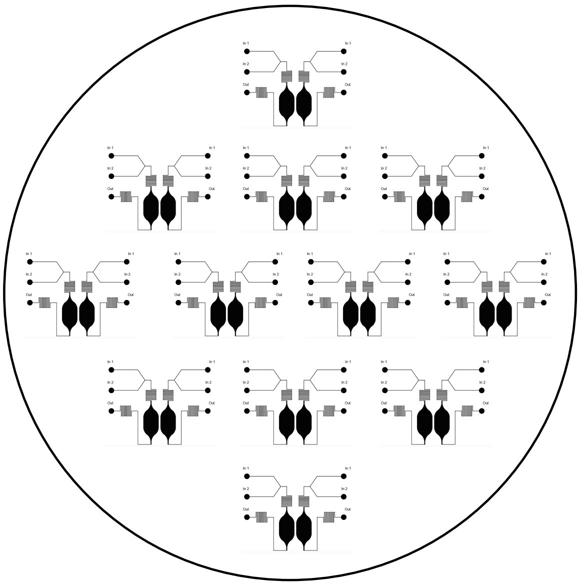

Supplementary Figure 7: Microfluidic platform n.1 (a) Photomask of the microfluidic device n.1 designed with CAD.

Suppl. Fig. 8

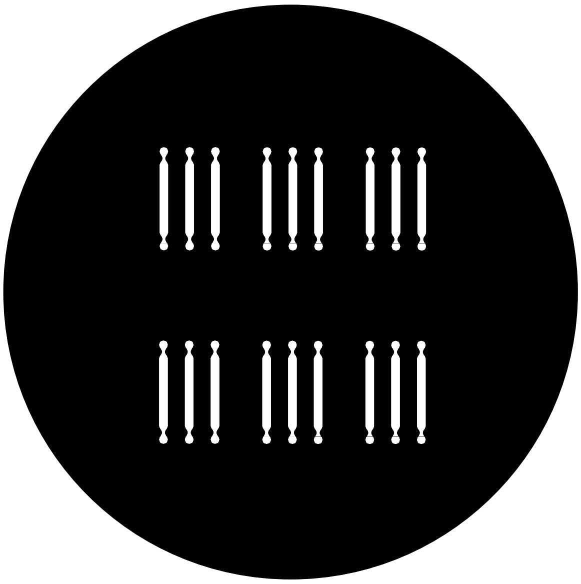

Supplementary Figure 8: Microfluidic platforms n.2. Photomask of the microfluidic device n.2 designed with CAD.

Supplementary Table 1

Name of gene	Source	Identifier
PER2	Thermo Fisher Scientific	HS 00256143_m1
BMAL1	Thermo Fisher Scientific	HS 00154147_m1
GAPDH	Thermo Fisher Scientific	Hs02758991_G1
