# Supplementary material for: Synchronization between peripheral circadian clock and feeding-fasting cycles in microfluidic device sustains oscillatory pattern of transcriptome
Source: Nat Commun. 2021 Oct 26;12:6185. doi: 10.1038/s41467-021-26294-9 (PMC8548598; doi:10.1038/s41467-021-26294-9)
Supplement: Supplementary file 3 — Description of Additional Supplementary Files [file 41467_2021_26294_MOESM3_ESM.docx]

Description of Additional Supplementary Files

Title: Supplementary Movie 1

Description: Video of time lapse of bioluminescence images captured every 30 min for 6.5 days from Per2::Luc fibroblasts integrated in a microfluidic chip composed by two parallel chambers. The cells in the chamber1 (left) are subjected to a medium change with a frequency of F=1/24 h^-1^, those in the chamber2 (right) are subjected to a medium change with a frequency of F=1 h^-1^.
